# Comparative and Spatial Transcriptome Analysis of *Rhododendron decorum* Franch. During the Flowering Period and Revelation of the Plant Defense Mechanism

**DOI:** 10.3390/genes15111482

**Published:** 2024-11-18

**Authors:** Weiwei Liu, Chenghua Yu, Kaiye Yang, Ling Wang, Zhongyu Fan, Xinchun Mo

**Affiliations:** 1 Lijiang Forest Biodiversity National Observation and Research Station, Kunming Institute of Botany, Chinese Academy of Sciences, Kunming 650201, China; liuweiwei@mail.kib.ac.cn (W.L.); yangkaiye@mail.kib.ac.cn (K.Y.); fanzhongyu@mail.kib.ac.cn (Z.F.); 2 School of Applied Technology, Lijiang Normal University, Lijiang 674199, China; 18468182962@163.com (C.Y.); wangling1014@163.com (L.W.)

**Keywords:** *Rhododendron decorum* Franch., comparative and spatial transcriptome analysis, KEGG pathway enrichment analysis, plant defense mechanism, flowering period

## Abstract

Background: *Rhododendron* is a globally distributed and extensive genus, comprising over 1000 species. In the southwestern mountains of China, there exists a remarkable diversity of *Rhododendron*, with Yunnan Province alone harboring more than 600 species. *R. decorum* Franch. has long been utilized by local communities for its medicinal and edible properties. However, the transcriptional regulation function, medicinal properties, and edibility characteristics of *R. decorum* Franch. currently lack a solid theoretical basis. Methods: Total RNA was extracted from leaves, corollas and androecium/gynoecium of *R. decorum* Franch. in Heqing county, followed by the construction of cDNA libraries and the de novo assembly of transcriptomes. Results: A total of 63,050 unigenes were extracted from the flowers and leaf organs of *R. decorum* Franch. Among these unigenes, 43,517 were predicted to be coding sequences, with 32,690 being effectively annotated. Differential gene expression enrichment was observed among different organs within their respective transcriptomes; notably floral organs exhibited significant defense against plant diseases along with signal transduction functions. Furthermore, during the flower harvesting period, all floral organs exhibited gene enrichment pathways associated with carbohydrate metabolism. Additionally, the stamen and pistil displayed flavonoid metabolism pathways, suggesting their potential applications as functional food or medicine. Conclusions: Our results shed light on plant–pathogen defense mechanisms and the molecular bias of flavonoids biosynthesis on flower organs during the flowering period, which might help to understand the consumption of *R. decorum* Franch. corollas by the Bai nationality of Heqing county.

## 1. Introduction

Rhododendrons, belonging to the genus *Rhododendron* within the family Ericaceae, are famous for its ecological, ornamental, and medicinal applications. Over 600 rhododendron varieties are distributed and primarily concentrated in the southwestern region of China, which serves as a globally renowned center for the distribution of rhododendrons [1]. *R*. *decorum* Franch., widely distributed throughout Yunnan Province in China and typically found at altitudes ranging from 1600 to 3500 m, is highly valued not only for its ornamental and medicinal properties but also as a dietary staple [2]. When growing at high altitudes, plants face many environment factors like the cold, water conditions, insect diets and pathogens, especially during the flowering period. Several studies have been conducted to uncover the stress tolerance mechanism of the *Rhododendron* genus, such as waterlogging and drought stress for *Rhododendron delavayi* Franch. [3,4], cadmium stress for *R. decorum* Franch. [5], cold stress for *Rhododendron aureum* Franch. [6], heat stress tolerance for *Rhododendron pulchrum* Sweet. [7,8] and salt tolerance for *Rhododendron simii* Planch. [9]. Researchers have tried to figure out the ecological adaptation of the *Rhododendron* genus to the alpine environment through stress tolerance mechanisms. However, they have primarily preferred to make conclusions on the individual plant organs such as the petals, leaves, or buds. The overview of the whole plant, including leaves and floral organs, as a constant defense mechanism is still unclear.

Transcriptome sequencing technology enables comprehensive and rapid acquisition of mRNA sequence information transcribed by specific tissues or organs in a particular state, facilitating the study of gene expression levels and structures at a global scale, thereby revealing molecular mechanisms underlying specific biological processes [10]. Through non-reference transcriptome sequencing technology based on second-generation sequencing platforms, large amounts of nucleotide sequences can be obtained to meet the requirement of covering the entire transcriptome [11,12]. Through de novo transcriptome sequence technology, it is possible to provide some insight into the flowers’ color formation and divergence of *Rhododendron* spp. [13,14,15,16]. Previous studies have compared transcriptomes of diverse organs under similar spatiotemporal conditions in economically important crops such as *Linum usitatissimum* (oilseed crop), *Brassica oleracea* var. *italica* (vegetable), *Machilus yunnanensis* (timber tree), and *Morus alba* (economic timber) [17,18,19,20]. Some *Rhododendron* species such as *R. decorum* Franch., *R. molle* G. Don and *R. micranthum* Turcz. have been reported to possess toxic properties harmful to both humans and livestock. However, of greater significance, these *Rhododendron* species were extensively utilized in traditional herbal remedies for addressing a wide range of ailments, including chronic bronchitis, coughing, rheumatic joint pain, osteomyelitis, severe cutaneous abscesses, gastric discomfort, nephritis, coagulation disorders and menstrual irregularities [21]. Although, the medicinal properties of the plant were described by researchers in the past, there is still insufficient research on the transcriptional regulation differences, physiological characteristics during the flowering period, and defense mechanisms against pathogens among different organs of *Rhododendron* species, particularly the corolla which holds potential for medicinal and edible applications.

The involvement of EF-hand family proteins in plant stress reflects a complex regulatory network governing the expression of multiple genes, as evidenced in several species [22,23,24,25]. Under different conditions, the EF-hand family proteins were found to activate different gene expression pattern responses to stress. For example, in cold conditions, the calcium-dependent protein kinase (CPK) and mitogen-activated protein kinase (MAPK) related to EF-hand family proteins were induced by the increased Ca^2+^ level in the plant membrane fluidity in response to the cold stress [26]. The MAPK cascade, which is highly conserved among eukaryotes [27], comprises three components: MAPK kinase kinases (MAPKKKs), MAPK kinases (MKKs), and MAPKs. In response to low temperatures, an elevation in Ca^2+^ levels occurs, leading to the activation of calcium sensors such as calcineurin B-like protein (CBL), calcium-dependent protein kinase (CPK), calmodulin (CaM), and Ca^2+^/CaM-dependent protein kinase (CCaMK) for signal transmission [28]. Thus, the plant defense mechanism of *R. decorum* Franch. in Heqing county during its flowering period is worth investigating, especially for the corollas which are regarded as a seasonal food for health care.

In this study, we conducted a comparative transcriptome analysis of different organs of *R. decorum* Franch. during its flowering period in Heqing county. Also, we explored EF-hand family proteins in the *R. decorum* Franch. plant–pathogen pathway that are specifically altered in response to environmental stress at the transcriptome levels. These results provide a basis for us to better understand the response mechanisms of *R. decorum* Franch. to environmental stress in its distribution area, and to also provide new insight into the relationship between the biosynthesis of components in the plant defense mechanism and the local people’s consumption.

## 2. Materials and Methods

### 2.1. Sample Collection and RNA Extraction

Fresh leaves and flowers corresponding to five accessions of *R. decorum* Franch. were collected from Heqing County (HQ) (100.075720° E, 26.485620° N, Altitude 3038 m), Dali Bai Autonomous Prefecture, Yunnan Province in May 2024. To evaluate the diversity between different floral organs, three to five intact flowers of the eugenic *R. decorum* Franch. plant were carefully collected from distinct populations and then separated into corolla (HQO), androecium/gynoecium (HQI) and whole flower (HQH). Additionally, three to five healthy leaves (HQL) were collected from the same individual. After that, all the collected leaves, corolla and androecium/gynoecium were pooled together, divided into three sequencing samples and immediately flash-frozen in liquid nitrogen. The frozen samples (named as HQO, HQI, HQH and HQL) were stored at −80 °C for transcriptomic analysis until use. The Qiagen RNeasy Plant Mini Kit (Qiagen company, Hilden, Germany) was utilized to isolate the total RNA from approximately 5 g of each sample according to the manufacturer’s instruction.

The tissues were ground into powder under liquid nitrogen and then mixed with preheated CTAB lysis reagent (containing 2% β-mercaptoethanol) at 65 °C. After a 15 min incubation at 65 °C followed by cooling to room temperature, the mixture was centrifuged at 4 °C with 12,000× *g* for 5 min. The resulting supernatant was transferred to another new tube. To further purify the RNA sample, chloroform/isoamyl alcohol (24:1) solution in a volume equal to that of the CTAB lysis buffer was added and mixed with the supernatant. This mixture underwent centrifugation at 4 °C with 12,000× *g* for 10 min. The clear upper aqueous layer was transferred to a new tube and combined with approximately two-thirds of the volume of isopropanol. The mixture was gently mixed by inversion and then placed at a temperature of −20 °C for a duration of 2 h. RNA precipitation was obtained from the mixture by centrifugation at 4 °C under 12,000× *g* centrifugation for 20 min. The supernatant was discarded, and the precipitation underwent resuspension followed by washing with 1 mL of solution containing 75% ethanol. After the washing step, the precipitation underwent another round of centrifugation at 4 °C with 17,500× *g* for 3 min. The supernatant was removed, and the precipitation was air-dried in the biosafety cabinet for approximately 3–5 min. Finally, DEPC-treated or RNase-free water (ranging from volumes between 20 to 200 µL) were added to dissolve the RNA.

The quantity and quality of the extracted total RNA were detected by using a NanoDrop 2000 spectrophotometer (Thermo Fisher Scientific Co., Ltd., Waltham, MA, USA) and an Agilent 2100 Bioanalyzer (Agilent Technologies, Santa Clara, CA, USA). The RNA samples with a minimum RNA Integrity Number (RIN) value of ≥8.0 and a 260/280 ratio ranging between 1.8 and 2.0 were chosen for subsequent cDNA library construction and sequencing [29].

### 2.2. cDNA Library Construction and Transcriptome Sequencing

For each sample, 1 μg total RNA was used for cDNA library construction, and the NEBNext^®^Ultra™ RNA Library Prep Kit from Illumina^®^ (NEB, Ipswich, MA, USA) was utilized to generate cDNA libraries following the manufacturer’s recommendations. Firstly, mRNA was purified from total RNA, and the first strand was synthesized by using the random hexamer primers and M-MuLV Reverse Transcriptase in NEBNext First Strand Synthesis Reaction Buffer (5X). Then, the second-strand cDNA synthesis was carried out with DNA Polymerase I and RNase H enzymes. When the synthesized cDNA fragments were approximately 150 bp in length, they were further purified by using the AMPure XP system (Beckman Coulter, Beverly, MA, USA). The blunt-ended cDNA fragments were added to an ‘A’ base at the 3’end, followed by ligation of adapters containing a ‘T’ nucleotide overhang at the 3’end to generate paired-end libraries for sequencing. The PCR amplification and enrichment of the library were conducted with adaptor region-based primers. The sequencing library’s quality was evaluated on the Agilent Bioanalyzer 2100 system (Agilent Technologies, Santa Clara, CA, USA). The libraries were sequenced on the Illumina HiSeqXTM10 (Illumina, Inc., San Diego, CA, USA) at Biomarker Technologies Co., Ltd. (Beijing, China).

### 2.3. Transcriptome Assembly and Annotation

The clean reads were obtained by trimming the adapter and removing low-quality reads from the raw reads generated by the cDNA library sequencing. Then, they were de novo assembled using the Trinity software (version 2.14.0) to generate the transcripts [30]. All the transcripts were further analyzed and the redundancies removed to acquire the unigenes without redundancy. The non-redundant unigenes were subjected to blast analysis against major public databases, including the NCBI non-redundant protein sequence database (NR database) (https://www.ncbi.nlm.nih.gov/protein/, accessed on 1 July 2024) [31], Swiss Prot database (https://www.uniprot.org/uniprot/, accessed on 1 July 2024) [32], Clusters of Orthologous Groups (COG) (http://www.ncbi.nlm.nih.gov/COG/, accessed on 1 July 2024) [33], Clusters of Protein homology (KOG) (https://ftp.ncbi.nih.gov/pub/COG/KOG/, accessed on 1 July 2024) [34], eggNOG4 (http://eggnogdb.embl.de/, accessed on 1 July 2024) [35], and Pfam (https://www.ebi.ac.uk/interpro/, accessed on 1 July 2024) [36], to identify homologous sequences with a cut-off value below 10^−5^. Gene Ontology (GO) (http://www.geneontology.org/, accessed on 1 July 2024) was employed to further categorize by Blast2GO software (version 2.5) with default parameters [37]. Furthermore, to obtain the corresponding amino acid sequence of unigenes, the TransDecoder software (v5.0.0) (https://transdecoder.github.io/, accessed on 1 July 2024) was used to predict the coding region sequence (CDS) based on aligning the protein domain sequence in the Pfam database.

### 2.4. Functional Annotation and Differential Expression Analysis

For functional annotation of the assembled unigenes, the entire identified unigenes were retrieved from the Kyoto Encyclopedia of Genes and Genomes (KEGG) database (http://www.genome.jp/kegg/, accessed on 1 July 2024). The threshold *E*-value was set below 10^−5^ by using the BLASTX. Also, the KEGG Orthology-based Annotation System (KOBAS) software (version 2.0) were employed to assign the unigenes into the KEGG orthology (KO) to figure out the biosynthesis pathway with default parameters [38].

Gene expression levels were calculated by using RNA-Seqby Expectation-Maximization (RSEM) software (version 1.3) to estimate the values of fragments per kilobase per million fragments mapped (FPKM) [39]. The DESeq2 software (version 1.30.1) was utilized for performing differential expression analysis based on the negative binomial distribution. The resulting *p* values were adjusted using Benjamini and Hochberg’s approach to control the false discovery rate, with a significance threshold of adjusted *p*-value < 0.05 assigned for identifying differentially expressed genes (DEGs) [40].

### 2.5. Simple Sequence Repeats (SSR) Identification

The unigenes derived from transcriptome sequences of leaves, corolla and androecium/gynoecium tissue were searched using the Microsatellite Searching Tool (MISA) (version 1.0) for the identification of SSR motifs. In this study, microsatellites ranging from mono-nucleotide to hexa-nucleotide were detected, and both perfect repeats (containing a single repeat motif) and compound repeats (containing two or more motifs separated by 100 base pairs) were identified [41].

## 3. Results

### 3.1. Transcriptome Sequencing and De Novo Assembly

Three cDNA libraries were constructed from the total RNA derived from the leaves and floral organs of *R. decorum* Franch. in Heqing county. After sequencing the constructed libraries on the Illumina HiSeq 2000 platform, approximately 89.90 Gb of clean data (0.3 billion reads) were generated. The quality assessment of the raw reads revealed that 93.12% of the reads had a base quality exceeding Q30 and were trimmed prior to assembly (Table S1). After removing the adapter, low quality reads and short reads (<50 bp), over 22 million high quality reads were obtained from the floral organs and leaves libraries, respectively. Furthermore, we performed the de novo assembly and removed the redundant clusters by using the Trinity software (version 2.14.0) to obtain a total of 63,050 unigenes with an average length of 971 bp, an N50 length of 1765 bp and GC content of 48.19% (Table S2). Over 50% (32,909) of the assembled unigenes were longer than 500 bp, and 31.33% (19,757) were longer than 1000 bp. The length of most unigenes fell between 200 bp and 2000 bp, as shown in Figure S1A. Additionally, a total of 43,517 coding sequences (CDSs) with average length 666 bp were predicted, including 19,671 (45.20%) complete CDSs. Among them, 17,113 (39.32%) CDSs were longer than 500 bp in length (Figure S1B). The sequence results suggested that the sequences were highly qualified and favored subsequent functional annotations.

### 3.2. Functional Annotation

To address the functional annotation of the assembled unigenes, the identified unigenes were blasted against the eight public databases for sequence similarity. Results showed that more than half of the unigenes (32,690, 51.85%) had significant matches in these databases, while others were uninformative. Among these annotations, the maximum annotation (49.93%) was observed against the NR database, while the COG had the least number of annotated unigenes (11.66%), and the KOG database resulted in 24.45% unigenes. Also, over 30% unigenes acquired significant hits from five databases, such as eggNOG (38.94%), GO (34.34%), Pfam (33.28%), Swissprot (30.95%) and KEGG (30.31%) (Table 1).

**Figure 1:**
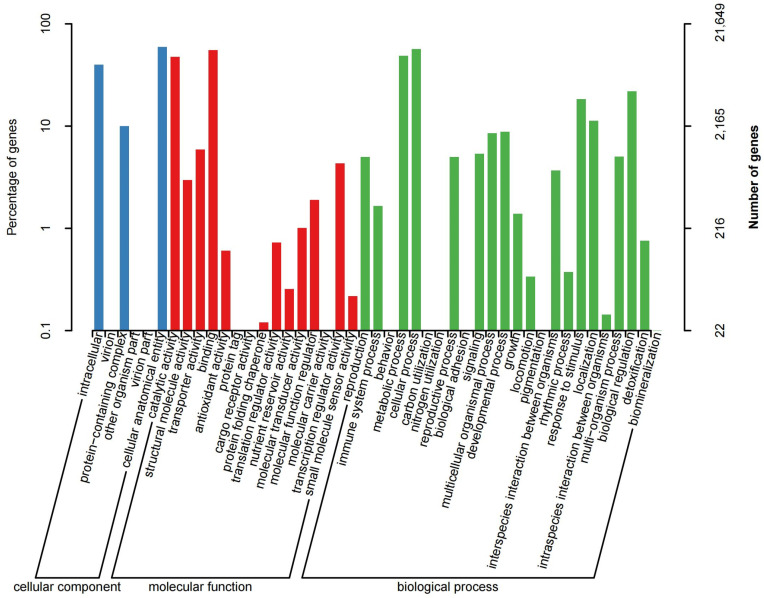
GO functional classifications of *Rhododendron decorum* Franch. unigenes.

Furthermore, the Nr identity distribution and *E*-value were estimated to evaluate the BLAST results. Statistical analysis proved that nearly three-fifths (59.41%) of mapped sequences represented a noticeable homology (<1.0 × 10^−50^), while the rest of the unigenes had *E*-values ranging from 1.0 × 10^−50^ to 1.0 × 10^−11^ (Figure S2A). Additionally, most of the mapped unigenes (84.26%) exhibited a similarity above 60%. Among them, 40.99% unigenes showed a similarity of >90%, and only 8.31% unigenes were below 50% (Figure S2B). The high identity along with a significant *E*-value demonstrated the reliability of de novo assembly quality. Sequence comparison of three homologues species showed that over half of unigenes (55.71%) exhibited the best hits to those of *Rhododendron williamsianum* Rehder & E. H. Wilson, while 13.13% unigenes were matched to *Camellia sinensis* and 11.38% unigenes to *Actinidia chinensis* var. (Figure S2C). Furthermore, GO annotation was conducted to facilitate the functional classification of the unigenes. Results showed that the entire estimated unigenes were classified into three major classes as biological process, cellular component and molecular function. In total, 21,649 unigenes were identified from sequenced HQI, HQO, HQH and HQL groups based on the sequence similarity and classified into 45 functional groups (Figure 1). The biological process category was subdivided into 24 classes, in which the predominant groups were cellular process (12,282 unigenes, 56.73%) and metabolic process (10,567 unigenes, 48.81%), followed by response to stimulus (3962 unigenes, 18.3%) and localization (2447 unigenes, 11.3%), indicating that the defense response mechanism to stimulus was active during the flowering development. In cellular component, most unigenes (12,873 unigenes, 59.46%) were involved in the cellular anatomical entity group. The second group was intracellular with 8634 unigenes accounting for 39.88%. Within the molecular function category, binding (12,007 unigenes, 55.46%) and catalytic activity (10,292 unigenes, 47.54%) were the two predominant groups in terms of quantity.

### 3.3. KEGG Analysis of Unigenes

**Figure 2:**
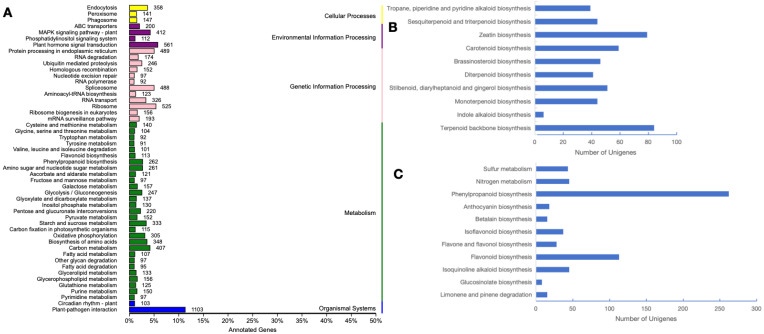
KEGG annotation of *R. decorum* Franch. unigenes. (**A**) KEGG functional classification of assembled unigenes. (**B**) Classifications of subcategory “metabolism of terpenoids and polyketides”. (**C**) Classifications of the subcategory “biosynthesis of other secondary metabolites”.

Genes collaboration within the same pathway is vital for their effective execution of biological functions. The adoption of a pathway-centric analysis approach facilitates the comprehension of their biological functions and the identification of unigenes associated with diverse biosynthetic pathways. Here, we found that a total of 14,874 unigenes were significantly matched into 136 KEGG pathways and divided into five primary categories, including cellular processing, environmental information processing, genetic information processing, metabolism and organismal systems (Figure 2A). Interestingly, the highest numbers of unigenes were annotated into “plant–pathogen interaction (1103 unigenes)”, suggesting that the plant was concerned with anti-pathogens during the floral development period, followed by the “plant hormone signal transduction (561 unigenes)” and “ribosome (525 unigenes)”, respectively. Also, the unigenes analysis contributed to the category of the “metabolism of terpenoid and polyketides”; 493 unigenes were found to fall into this category. The largest group of unigenes (84 unigenes) were involved in the subcategory “terpenoid backbone biosynthesis”, followed by the “zeatin biosynthesis” (79 unigenes). Six subcategories had 40 to 60 unigenes except the subcategory “indole alkaloid biosynthesis”, which had only 6 unigenes (Figure 2B). When exploring the unigenes correlated to the secondary metabolism, 629 unigenes were found to be involved in 11 pathways with over demisemi of them belonging to the pathway “phenylpropanoid biosynthesis (262 unigenes)”, followed by the “flavonoid biosynthesis” pathway (113 unigenes) (Figure 2C).

### 3.4. Differential Expression Genes Identification and Expression Pattern

**Figure 3:**
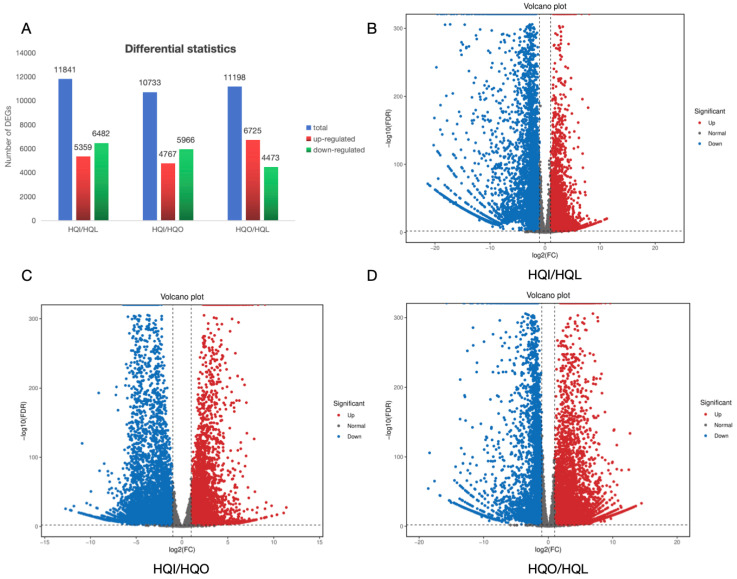
Statistics of differential expressed genes and comparisons between different groups. (**A**) Differentially expressed genes in different compared groups. The horizontal axis represents distinct sets of differentially expressed genes, where blue color represents the differential expressed genes in different groups, red color indicates upregulated, and green signifies downregulated. Meanwhile, the vertical axis corresponds to the number of differential expressed genes. (**B**) Volcano plot of differentially expressed genes in HQI/HQL. (**C**) Volcano plot of differentially expressed genes in HQI/HQO. (**D**) Volcano plot of differentially expressed genes in HQO/HQL. Each point on the graph represents a gene, with the horizontal axis indicating the logarithmic-fold change in gene expression between two samples. The vertical axis represents the negative logarithm of the false discovery rate. Blue points represent downregulated differentially expressed genes, red points represent upregulated differentially expressed genes, and gray points represent non-differentially expressed genes.

To explore the genes involved in regulating the floral organ development of *R. decorum* Franch. in HQ county, the differential expression between different floral organs was analyzed. Results showed that a total of 33,772 genes were significantly differently expressed among three groups: 11,841 genes in the HQI/HQL group, 10,733 genes in the HQI/HQO group and 11,198 genes in the HQO/HQL group. In the HQI/HQL and HQI/HQO groups, they shared the same gene expression patterns with 5359 and 4767 DEGs upregulated and 6482 and 5966 downregulated, respectively. However, the HQO/HQL showed the opposite expression pattern with the other two groups, with 6725 upregulated and 4473 downregulated (Figure 3).

### 3.5. Enrichment Analysis of DEGs in Different Organs Comparison

**Figure 4:**
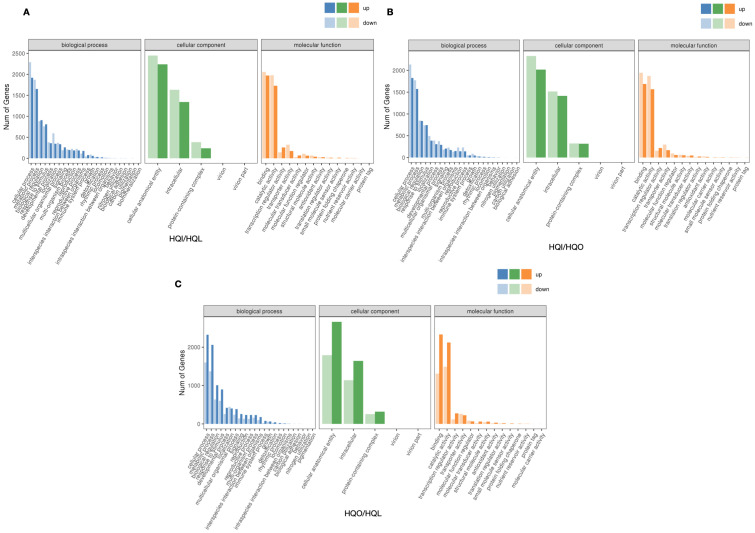
GO enrichment analysis of differential expression genes within different organ comparison groups. (**A**) GO terms of DEGs in HQI/HQL group. (**B**) GO terms of DEGs in HQI/HQO group. (**C**) GO terms of DEGs in HQO/HQL group.

Functional enrichment analysis was employed to estimate the potential functions of differential expression genes (DEGs) between the HQI/HQL, HQI/HQO and HQO/HQL group, respectively. Within the HQI/HQL group, 7675 DEGs were matched to 43 GO terms, of which eight terms were significantly enriched (Figure 4A). In the HQI/HQO group, 7117 DEGs were dropped into 45 GO terms, and six of them were significantly enriched (Figure 4B). Lastly, 7255 DEGs were found enriched in GO terms, and eight terms were significantly enriched (Figure 4C). Within these three groups, they all shared the same trends in the most significantly enriched terms of ‘cellular anatomical entity’, ‘cellular process’ and ‘binding’, respectively.

KEGG enrichment analysis of DEGs between different organs of *R. decorum* Franch. from HQ county suggested that 20,175 DEGs were assigned into 135 KEGG pathways. Among them, they were observed to show significant upregulation and downregulation in different comparison groups exhibiting distinct differential expression patterns (Table 2). In the HQO/HOL group, the significantly upregulated DEGs were predominantly enriched in KEGG pathways such as plant–pathogen interaction, plant hormone signal transduction, starch and sucrose metabolism, photosynthesis and photosynthesis-antenna proteins (*q*-value < 0.001). The significantly downregulated DEGs were mainly enriched in pathways including oxidative phosphorylation, terpenoid backbone biosynthesis, and proteasome. The results suggest that the corolla was focused on the photosynthesis and accumulated the starch and sucrose to sustain floral development, while the leaves were occupied with the biosynthesis of insect-resistant-related chemicals. In the HQI/HOL group, the trends of significantly upregulated DEGs were shown the same as in the HQO/HQL group, except for the flavonoid biosynthesis and MAPK signaling pathway which were highly enriched in the HQI/HQL group, suggesting that the androecium/gynoecium were underdeveloped with the synthesis of the polysaccharide and synthesized the flavonoids to prevent insects. The significantly downregulated DEGs in this group were mainly enriched in glycerophospholipid metabolism and the phosphatidylinositol signaling system pathway. In the HQI/HQO group, only three significant KEGG pathways were found where two upregulated pathways were enriched in plant hormone signal transduction and proteasome, while the downregulated pathway in glycerophospholipid metabolism indicated that the androecium/gynoecium were in the process of floral organ maturation, and the corolla response was in defense against the environment.

### 3.6. DEG Expression Patterns of the EF-Hand Protein Family in Plant–Pathogen Interaction Pathways

**Figure 5:**
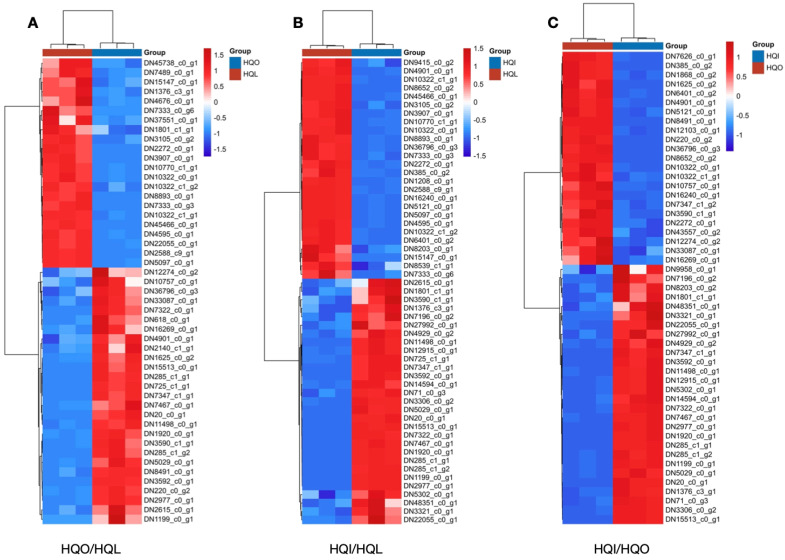
The heatmaps of DEG expression patterns in different groups of *R. decorum* Franch. (**A**) DEG expression patterns in HQO/HQL group. (**B**) DEG expression patterns in HQI/HQL group. (**C**) DEG expression patterns in HQI/HQO group. Clustering plot depicting the differential expression analysis of genes belonging to the EF-hand protein family. Blue color represents the downregulated and red color the upregulated.

EF-hand protein families play a pivotal role in the transmission of calcium signals in plants, which mostly respond to the plant–pathogen interaction, plant growth and development. The key EF-hand proteins encompass calmodulins (CaMs), calmodulin-like proteins (CMLs), calcineurin B-like proteins (CBLs), and calcium-dependent protein kinases (CDPKs/CPKs). To explore the plant–pathogen interaction by EF-hand protein families during the floral development of *R. decorum* Franch., the EF-hand-related unigenes were identified and compared within different groups to figure out their expression patterns with FPKM values. The selected threshold value was a *p*-value < 0.05. Over 50 DEGs were identified in each group with 50 DEGs in the HQO/HQL group, 59 DEGs in HQI/HQL and 55 DEGs in HQI/HQO (Figure 5). The results showed that the EF-hand proteins had different expression patterns in leaf, corolla and androecium/gynoecium, which indicate that the Ca^2+^-related genes expression patterns within different floral organs might respond to the defense against the environment effect like insect intake, microbial pathogen infection and other stresses. In the HQO/HQL group, 22 DEGs were found upregulated at the corolla, where 28 DEGs showed regulations at the leaf, suggesting that the two organs were sensitive to the environment in the process of floral development (Table S3). The same trends of regulated DEGs were observed in the HQI/HQL group (Table S4). In the HQI/HQO group, the numbers of upregulated DEGs in the corolla were greater than that in the androecium/gynoecium, indicating that the corolla might be more attractive to insects or sensitive to environmental changes (Table S5).

## 4. Discussion

The *R. decorum* Franch. species is widely distributed in Yunnan Province, China and highly regarded by locals as a seasonal functional food that can be found in local markets. A comprehensive transcriptome sequencing of leaves and floral organs of *R. decorum* Franch. from Heqing county resulted in a high-quality dataset of 89.90 Gb, yielding an impressive total of 63,050 unigenes with an average length of 971 bp, N50 length of 1765 bp and GC content of 48.19%, indicating exceptional assembly completeness. Among them, 32,690 genes were annotated, resulting in an annotation rate of 51.85%, which is slightly higher than that reported in other studies on related plants. For instance, a total of 92,463 genes with functional annotations for 38,724 genes and an annotation rate of 41.88% were reported for the species *R. obtusum* (Lindl.) Planch. [42]. Similarly, in the de novo transcriptome assembly of *R. latoucheae* Franch., 80,660 genes were identified with functional annotations for 34,867 genes and an annotation rate of 43.22% [43]. Also, the transcriptome sequence of *R. molle* (Blume) G. Don uncovered a total of 66,026 genes with functional annotations for 31,298 genes and achieved an annotation rate of 47.40% [10]. The annotation of specific functional genes in this species, along with those in other species within the same genus, has significantly contributed to our understanding of this valuable plant resource. Simultaneously, it has played a pivotal role in advancing future research endeavors aimed at unraveling the biological and ecological characteristics of this species, as well as facilitating resource assessment and development.

Transcriptional expression often varies across different plant organs, which holds significant implications for the edibility, medicinal properties, and biological defense mechanisms of plants. Glucoraphanin, a widely recognized phytochemical compound found in broccoli, has demonstrated that the transcriptional expression patterns of this chemical can affect its predominant accumulation in small flowers [18]. Furthermore, the research conducted on the species *Baphicacanthus cusia* (Nees) Bremek. towards exogenous stimuli has also highlighted variations in organ-specific responses [44]. Similarly, the same phenomena were found where the hormone signaling pathways were highly enriched across different organs during an experimental simulation of cadmium stress recovery in mung beans [45].

Here, we also found that the genes were observed to be organ-specific among different organs. KEGG pathway enrichment analysis revealed that significant differential expression patterns were observed between floral organs (corolla and androecium/gynoecium). Corollas exhibited stronger energy metabolism functions in pentose and glucuronate interconversions as well as glycerophospholipid metabolism, indicating that the corollas exhibited enhanced saccharides production capacity during the flowering period [46]. The androecium/gynoecium preferred to enhance proficiency in plant hormone signal transduction. Comparing the enriched KEGG pathways of differentially expressed transcripts within leaves and various floral organs, results suggest that the corollas appeared to have strong defense mechanisms (plant–pathogen interaction pathway), hormone signal transduction, and starch and sucrose metabolism. Additionally, the androecium/gynoecium also showed active flavonoid biosynthesis, suggesting that the flowers possessed strong biological defense activity against insects’ diets or pathogens during the flowering period. Further analysis proved that both corollas and androecium/gynoecium maintained high gene expression in the plant–pathogen interaction pathway to resist pathogenic infections. Furthermore, the androecium/gynoecium were also found to sustain the flavonoids synthesis to prevent insects’ diets. The flavonoids, a subclass of polyphenols, possessed the potential to exert pesticidal effects against plant pathogens and insects by actively participating in plant defense mechanisms [47]. Also, the same KEGG pathway enrichment in α-linolenic acid metabolism, benzoxazinoid biosynthesis, flavonoid biosynthesis and phenylpropanoid biosynthesis were observed in maize-involved defense reactions [48]. Additionally, a recent study on the adaptive molecular mechanisms of the *Rhododendron* genus revealed a substantial presence of exclusive gene families in these plants. These gene families exhibited significant enrichment in plant–pathogen interactions and fatty acid metabolism pathways. This genomic perspective corroborated the evidences of differential expression analysis on the transcriptome comparison within different organs of *R. decorum* Franch., where the plant–pathogen interaction pathways were normally enriched in flowers and multiple fatty acid metabolism pathways in leaves [49].

Furthermore, the analysis of the plant–pathogen interaction pathway revealed a significantly elevated expression level in EF-hand protein family genes found in all chosen organs. Differential gene expression pattern analysis across different organs further proved that the EF-hand protein family genes might play a key role in regulating the gene expression variations within organs. Calcium (Ca^2+^), as a ubiquitous second messenger, played a pivotal role in diverse signaling mechanisms across various life forms. As the life style transitioned from aquatic to terrestrial environments, the Ca^2+^ signal transduction system was immediately active to show significant expansion and diversification. Accumulated evidences within different organs of *R. decorum* Franch. suggest that Ca^2+^ modulates gene transcription by interacting with EF-hand proteins that serve as crucial mediators in plant-signaling events during the flower period [50,51]. In this study, we also found high expression patterns of EF-hand proteins in different organs when the floral development was still in the maturation process. The *R. decorum* Franch. needed to accumulate more active defense proteins to cope with the challenge of insects’ diets or other potential pathogen infections.

In summary, the comparative transcriptome analysis of different organs in *R. decorum* Franch. provided a comprehensive overview of the genetic basis underlying this species, which can be utilized for ornamental purposes, consumption, and ecological restoration as a native tree species. The results elucidated the intricate interplay between complex evolutionary forces and functional adaptations shaping its biology [52,53,54]. Extensive studies on species of *Rhododendron* have been conducted on stress resistance and flower color regulation [5,6,7,14]. However, there is still a scarcity of reports regarding its edibility and medicinal properties. By conducting a comprehensive de novo transcriptome assembly and comparative analysis of *R. decorum* Franch. from Heqing county, it held a great significance in establishing a theoretical foundation for its edibility, medicinal properties, as well as resource development and utilization. Additionally, the enrichment of sugar biosynthesis metabolism pathway in corollas of *R. decorum* Franch. suggested this plant might serve as a potential medicinal plant. Obviously, the local residents’ practice of removing the androecium/gynoecium before consuming *R. decorum* Franch. flowers was from experience in removing poisonous constituents to ensure the edibility without intake of flavonoids. The results of comparative transcriptome analysis of different organs in the flowering development period of *R. decorum* Franch. sheds light on the plant–pathogen defense mechanism and its molecular bias of flavonoids biosynthesis in the flower organs during the flowering period, which might help to understand the consumption of corollas of *R. decorum* Franch. by the Bai nationality of Heqing county.

## 5. Conclusions

The transcriptomes of various organs of *R. decorum* Franch. from Heqing county were sequenced and de novo assembled, resulting in a total of 63,050 unigenes with an average length of 971 bp obtained with a N50 length of 1765 bp and a GC content of 48.19%. Among them, 43,517 unigenes were predicted to be coded sequences and 32,690 unigenes were successfully annotated. Comparative transcriptomes analysis of different organs of *R. decorum* Franch. exhibited distinct enrichment patterns of differentially expressed genes during the flowering period. GO and KEGG pathway analysis of these enriched differentially expressed genes further revealed that significant enrichment was observed in the plant disease defense and signal transduction pathway, suggesting that *R. decorum* Franch. has a robust ability to resist diseases during the flowering and organ formation period. Moreover, the corollas of *R. decorum* Franch. displayed gene enrichment pathways related to carbohydrate metabolism, while the androecium/gynoecium exhibited flavonoid metabolism pathways, suggesting that the corollas of this species are a potential resource applied as functional food or medicine.

## Figures and Tables

**Table 1:** Annotation of identified unigenes against eight different databases.

Annotated Database	Annotated Number	Percentage of Annotated Genes
Nr	31,480	49.93%
eggNOG	24,550	38.94%
GO	21,649	34.34%
Pfam	20,980	33.28%
Swissprot	19,517	30.95%
KEGG	19,109	30.31%
KOG	15,417	24.45%
COG	7351	11.66%
All Annotated	32,690	51.85%

**Table 2:** KEGG enrichment analysis of differential expression genes across distinct groups.

The Primary Categorization of KEGG	The Secondary Categorization of KEGG	Pathway ID in KEGG	Designations of Pathways (or Subcategories) in the KEGG	Gene Ratio	Bg Ratio	Enrich Factor	Gene Num
**HQO/HQL**							
**Upregulation**							
Organismal Systems	Environmental adaptation	ko04626	Plant–pathogen interaction	15.39%	11.32%	1.36	306
Environmental Information Processing	Signal transduction	ko04075	Plant hormone signal transduction	7.70%	5.76%	1.34	153
Metabolism	Carbohydrate metabolism	ko00500	Starch and sucrose metabolism	6.09%	3.42%	1.78	121
Metabolism	Energy metabolism	ko00195	Photosynthesis	1.71%	0.79%	2.16	34
Metabolism	Energy metabolism	ko00196	Photosynthesis-antenna proteins	0.80%	0.19%	4.13	16
**Downregulation**							
Metabolism	Energy metabolism	ko00190	Oxidative phosphorylation	5.81%	3.13%	1.85	84
Metabolism	Metabolism of terpenoids and polyketides	ko00900	Terpenoid backbone biosynthesis	1.94%	0.86%	2.25	28
Genetic Information Processing	Folding, sorting and degradation	ko03050	Proteasome	1.87%	0.73%	2.56	27
**HQI/HQL**							
**Upregulation**							
Organismal Systems	Environmental adaptation	ko04626	Plant–pathogen interaction	18.94%	11.32%	1.67	318
Environmental Information Processing	Signal transduction	ko04075	Plant hormone signal transduction	10.18%	5.76%	1.77	171
Environmental Information Processing	Signal transduction	ko04016	MAPK signaling pathway–plant	6.37%	4.23%	1.51	107
Metabolism	Carbohydrate metabolism	ko00500	Starch and sucrose metabolism	5.36%	3.42%	1.57	90
Metabolism	Biosynthesis of other secondary metabolites	ko00941	Flavonoid biosynthesis	2.20%	1.16%	1.9	37
Metabolism	Energy metabolism	ko00195	Photosynthesis	1.97%	0.79%	2.49	33
Metabolism	Energy metabolism	ko00196	Photosynthesis-antenna proteins	0.83%	0.19%	4.28	14
**Downregulation**							
Metabolism	Lipid metabolism	ko00564	Glycerophospholipid metabolism	3.08%	1.60%	1.92	56
Environmental Information Processing	Signal transduction	ko04070	Phosphatidylinositol signaling system	2.25%	1.15%	1.96	41
**HQI/HQO**							
**Upregulation**							
Environmental Information Processing	Signal transduction	ko04075	Plant hormone signal transduction	9.07%	5.76%	1.58	146
Genetic Information Processing	Folding, sorting and degradation	ko03050	Proteasome	2.42%	0.73%	3.33	39
**Downregulation**							
Metabolism	Lipid metabolism	ko00564	Glycerophospholipid metabolism	3.03%	1.60%	1.89	49

**Note:** The KEGG enrichment pathways were present with a *q*-value < 0.001. The Gene Ratio metric quantifies the proportion of DEGs relative to differentially compared groups. The Bg Ratio represents the ratio of DEGs associated with a specific function to the total number of genes. The enrich factor refers to the ratio of pathway-annotated DEGs among DEGs compared to that among all genes. Gene Num represents the count of DEGs enriched within the pathway.

## Data Availability

The data presented in this study are available on request from the corresponding author. The data are not publicly available due to privacy.

## Supplementary Materials

The following supporting information can be downloaded at: https://www.mdpi.com/article/10.3390/genes15111482/s1, Figure S1. The length distribution of assembled unigenes and predicted CDSs. (**A**) Length distribution of assembled unigenes. (**B**) Length distribution of predicted CDSs.; Figure S2. Characteristics of homology search of assembled unigenes against the NR database. (**A**) E-value distribution of top Blast hits. (**B**) Similarity distribution of unigenes. (**C**) Species distribution of Blast hits; Table S1. Statistical evaluation for sample sequencing data; Table S2. The results of transcriptome sequencing data assembly; Table S3. The DEGs expression patterns of the EF-hand protein family in the HQO-HQL group; Table S4. The DEGs expression patterns of the EF-hand protein family in the HQI-HQL group; Table S5. The DEGs expression patterns of the EF-hand protein family in the HQI-HQO group.
