# Supplementary figures and images for: Comparative and Spatial Transcriptome Analysis of Rhododendron decorum Franch. During the Flowering Period and Revelation of the Plant Defense Mechanism
Source: Genes (Basel). 2024 Nov 18;15(11):1482. doi: 10.3390/genes15111482 (PMC11593350; doi:10.3390/genes15111482)

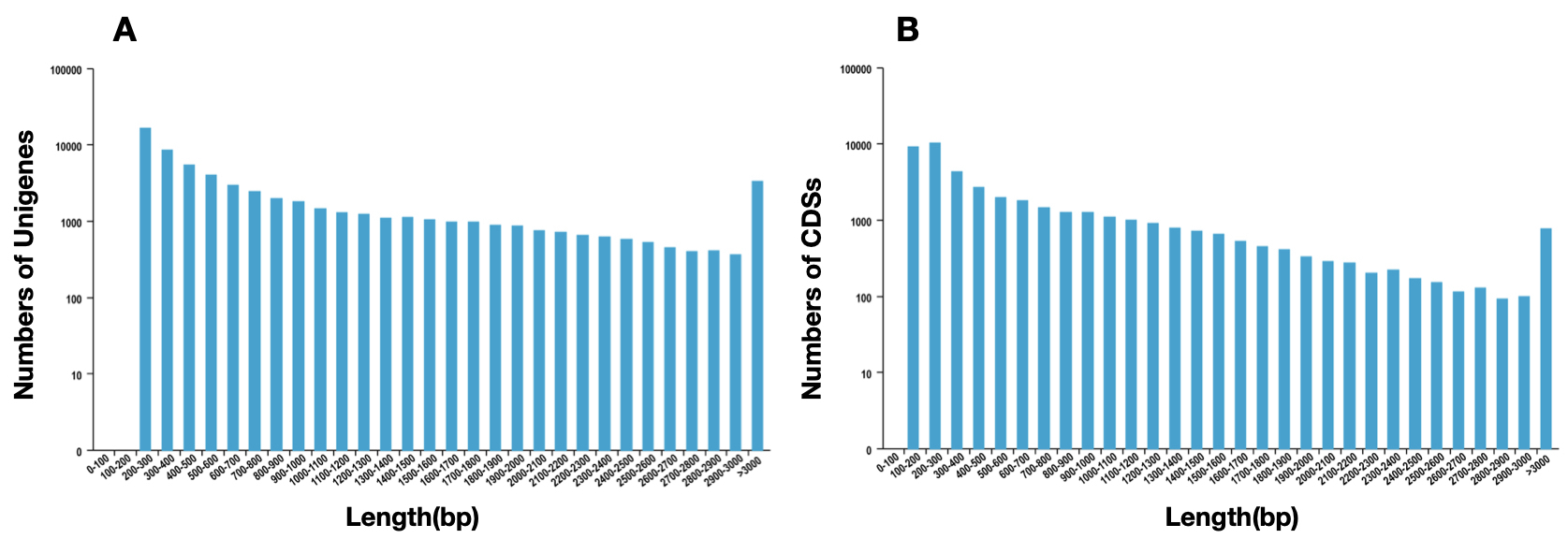

Supplement: Supplementary file 1 [file genes-15-01482-s001.zip › Figure S1 The Length distribution of assembled unigenes and predict CDSs.jpeg]

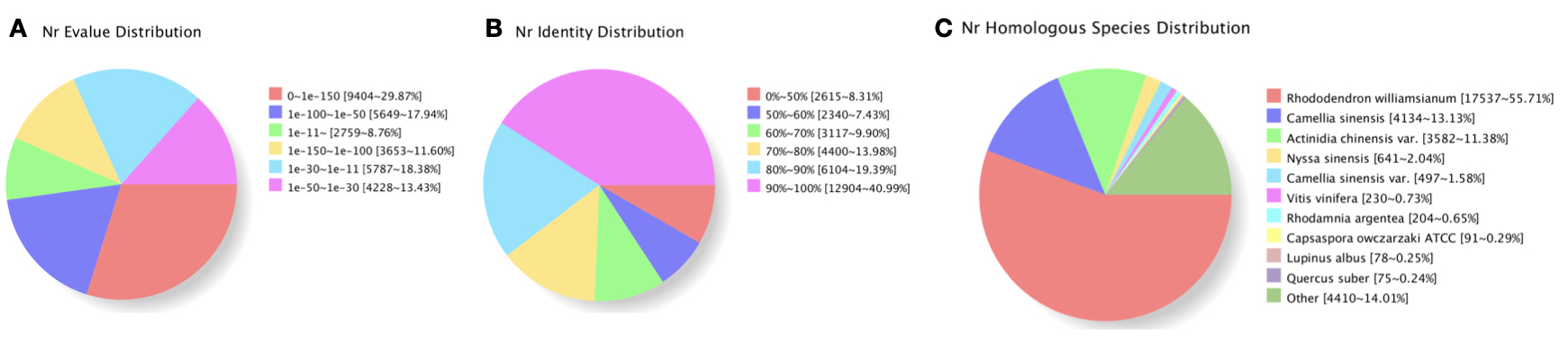

Supplement: Supplementary file 1 [file genes-15-01482-s001.zip › Figure S2 Characterstic of homolgy search of assembled unigenes against NR database.jpeg]
